# Supplementary material for: Targeted CRISPR-Cas9 screening identifies core transcription factors controlling murine haemato-endothelial fate commitment
Source: Nat Commun. 2025 Dec 13;16:11412. doi: 10.1038/s41467-025-66230-9 (PMC12738756; doi:10.1038/s41467-025-66230-9)

Supplementary Information

Targeted CRISPR-Cas9 screening identifies core transcription factors controlling murine haemato-endothelial fate commitment

Michael Teske[1,2], Tobias Wertheimer[1], Stefan Butz[2], Pascale Zwicky[1], Izaskun Mallona[3], Svenja L Nopper[1], Christian Münz[1], Ulrich Elling[4], Christophe Lancrin[5], Burkhard Becher[1], Ana Rita Grosso[6,7], Tuncay Baubec[2,8], Nina Schmolka[1,2,9*]

Contents

Supplementary Figures and Legends 1-8
Supplementary Table 1-3

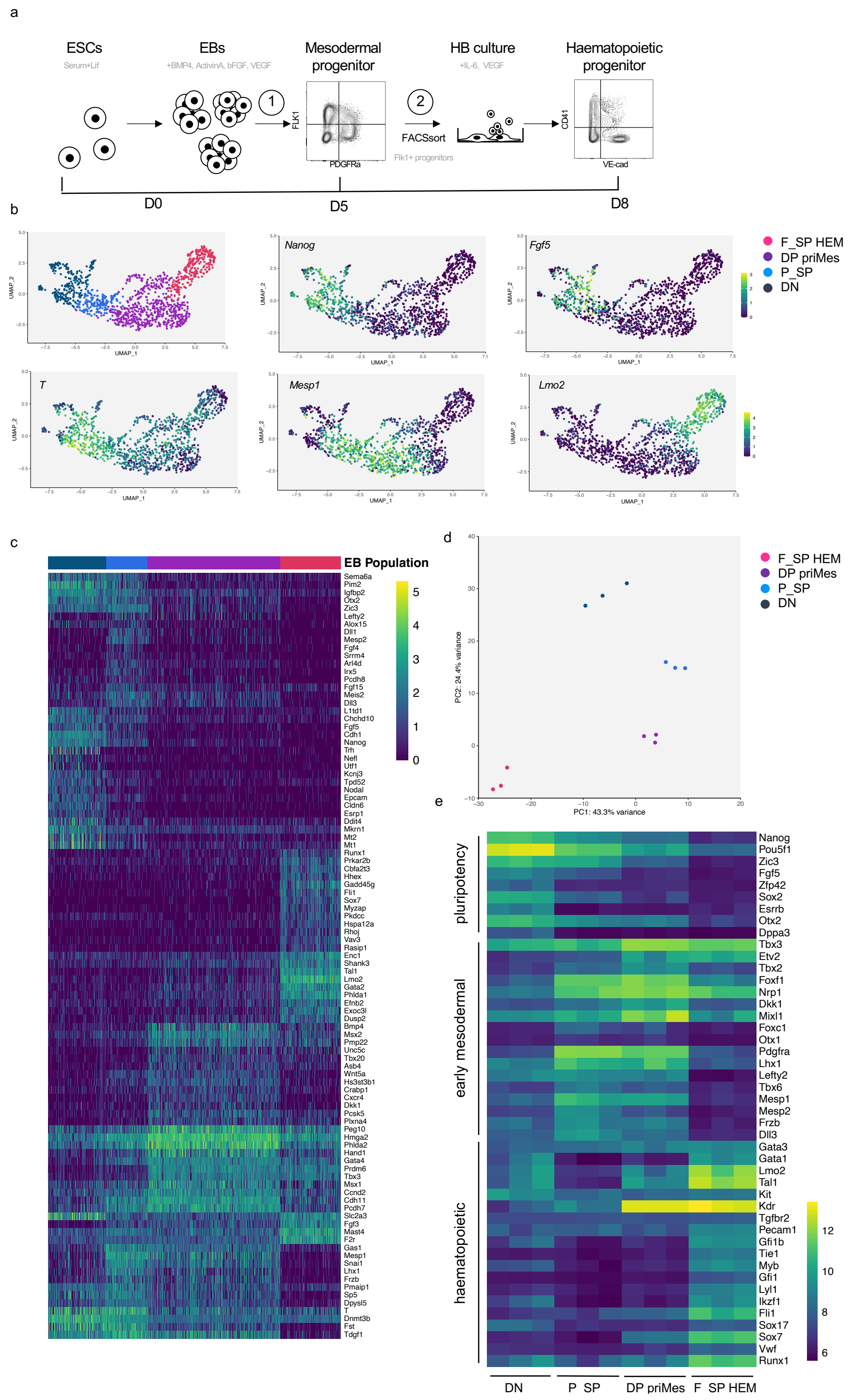

Supplementary Figure 1:
a Schematic of ESC-derived haematopoietic differentiation model. **b** Expression of selected mesodermal and haematopoietic signature genes in mesodermal populations analysed by scRNA-seq. **c** Heatmap indicating gene expression by scRNA-seq of top 20 differential regulated genes in each mesodermal population. **d** Principal component analysis of bulk RNA-seq data of mesodermal populations obtained from EB cultures at day5. Each point represents an individual culture. **e** Heatmap indicating gene expression by bulk RNA-seq of pluripotency-, mesoderm- and haematopoiesis-associated genes in the analysed mesodermal populations (VST values).

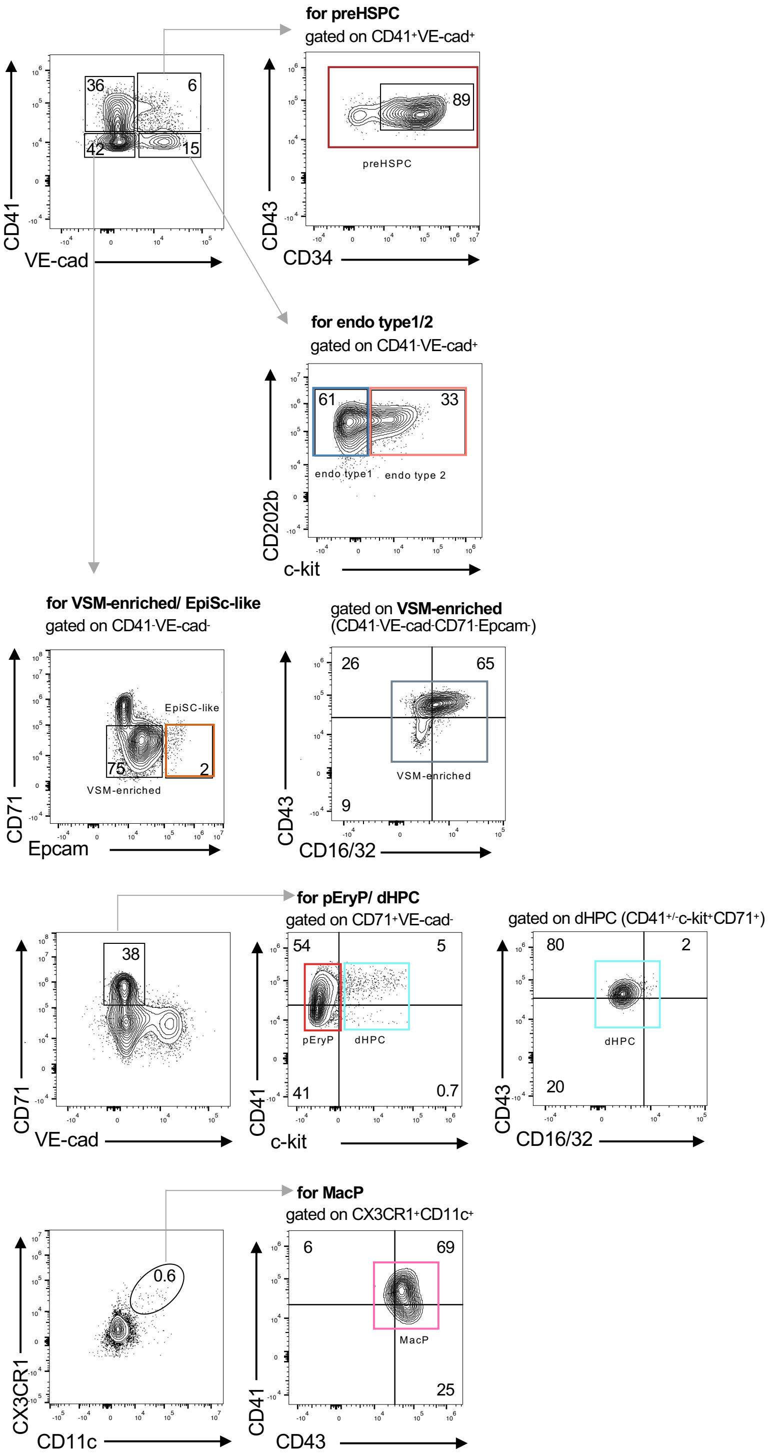

Supplementary Figure 2:
Representative FACS plots illustrating a conventional manual gating strategy of haemangioblast cultures analysed at day 2.5. The depicted manual gating strategy is solely to visualise cells as cellular clusters were defined by an unsupervised clustering approach, detailed in Methods section.

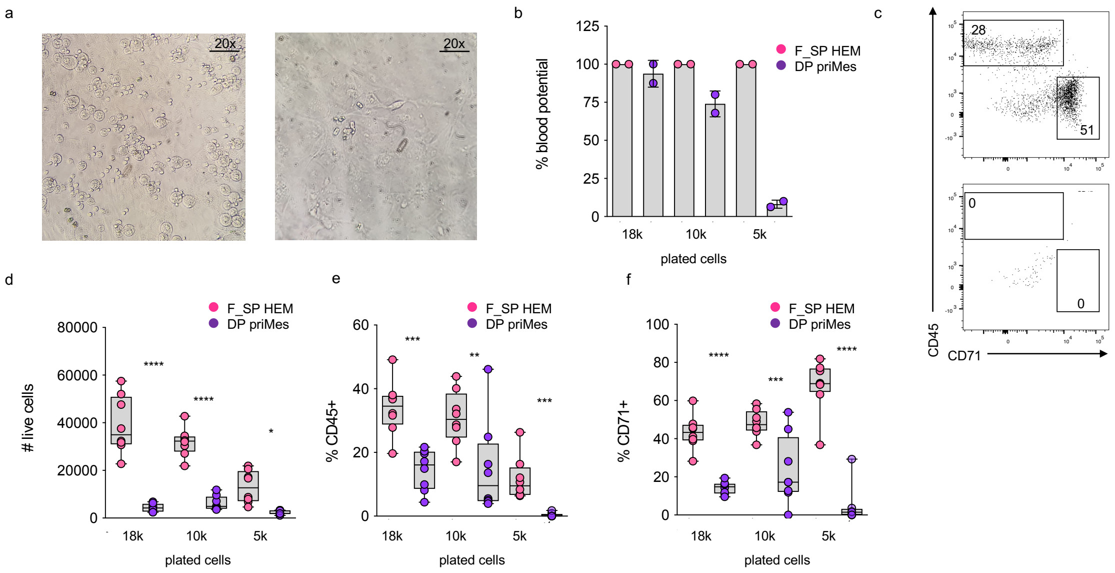

Supplementary Figure 3:
a Representative microscopy images of an in vitro haematopoietic culture containing haematopoietic cells started from F_SP HEM (left) and a haematopoietic culture without haematopoietic cells started from DP priMES (right) at 20× magnification at day 9.5. **b** Frequency of blood forming potential after 9.5 days of cultures started from varying cell numbers (18k, 10k, 5k) of F_SP HEM and DP priMes. **c** Representative flow cytometry analysis of CD45 and CD71 surface expression of a positive in vitro haematopoietic culture started from F_SP HEM (upper) and negative haematopoietic cultures started from DP priMES (lower) at day 9.5. (**d**) Number of live cells (F_SP HEM 18k vs. DP priMes 18k $p<0.0001$, F_SP HEM 10k vs. DP priMes 10k $p<0.0001$, F_SP HEM 5k vs. DP priMes 5k $p=0.0164$) and (**e**) percentage of CD45+ cells (F_SP HEM 18k vs. DP priMes 18k $p= 0.007$, F_SP HEM 10k vs. DP priMes 10k $p= 0.0083$, F_SP HEM 5k vs. DP priMes 5k $p=0.0009$) (**f**) and CD71+ cells (F_SP HEM 18k vs. DP priMes 18k $p= 0.008$, F_SP HEM 10k vs. DP priMes 10k $p= 0.0083$, F_SP HEM 5k vs. DP priMes 5k $p<0.0001$) from individual haematopoietic cultures started from F_SP HEM and DP priMes isolated from EB cultures at day 9.5. Each data point (in d- e) represents an individual culture n= 8. P-values were calculated using a one-way ANOVA multiple comparisons analysis. Box plot whiskers represent the minimum and maximum value. Bounds mark the 25th and 75th percentiles. Central line is the median. Source data for (**b, d-f**) are provided as a Source Data file.

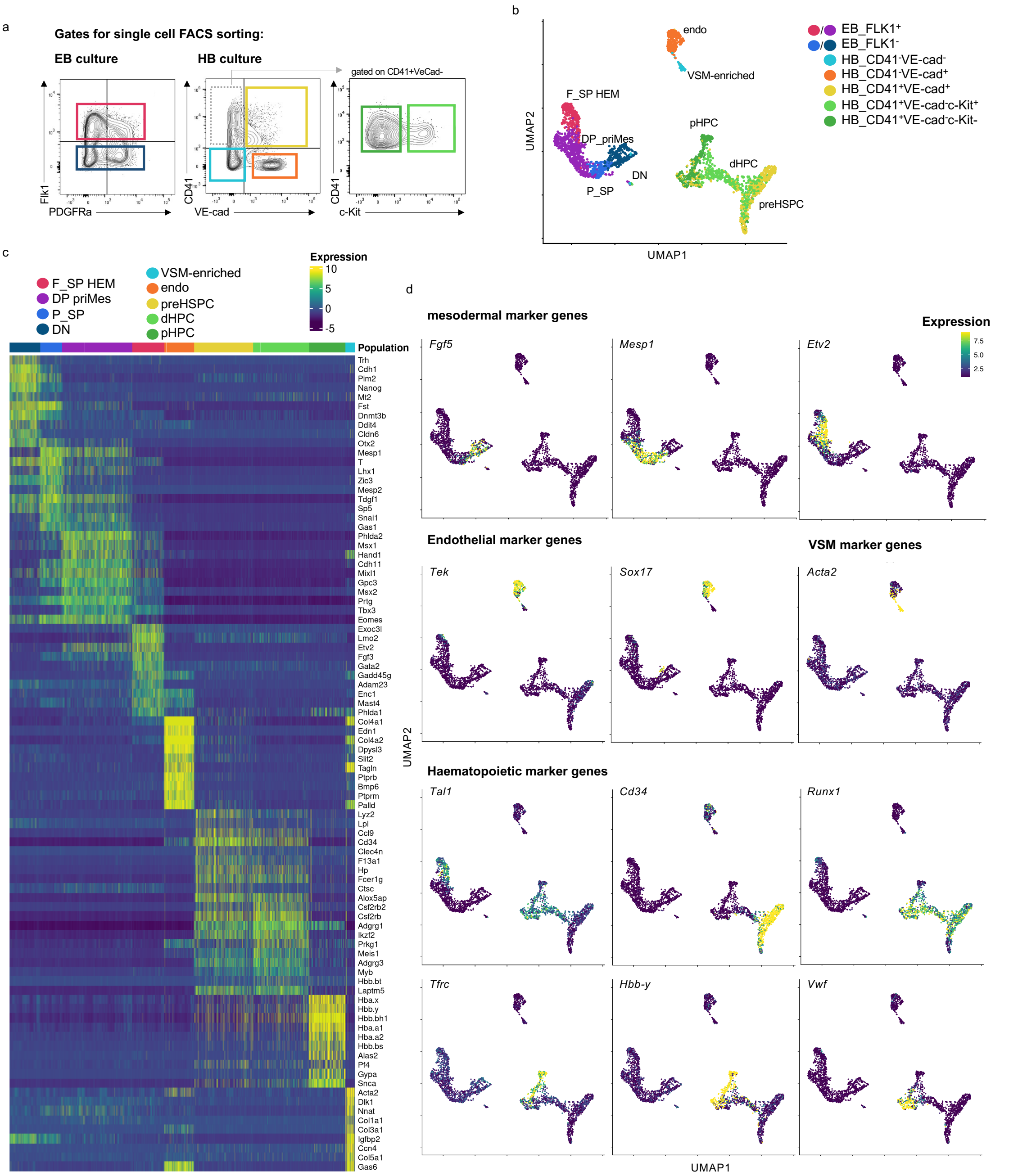

Supplementary Figure 4:
a Gating strategy of single cell FACS-sorting of EB and HB cultures. **b** UMAP of 2623 cells FACS-sorted from EB and HB cultures coloured by cell surface marker expression. **c** Heatmap indicating gene expression by scRNA-seq of top 10 marker genes of each cell cluster. **d** Expression of selected signature genes analysed by scRNA-seq. pHPC: primitive haematopoietic progenitor cells; dHPC: definitive haematopoietic progenitor cells; preHSPC: pre-haematopoietic stem progenitor cells; VSM: vascular smooth muscle; endo:endothelia.

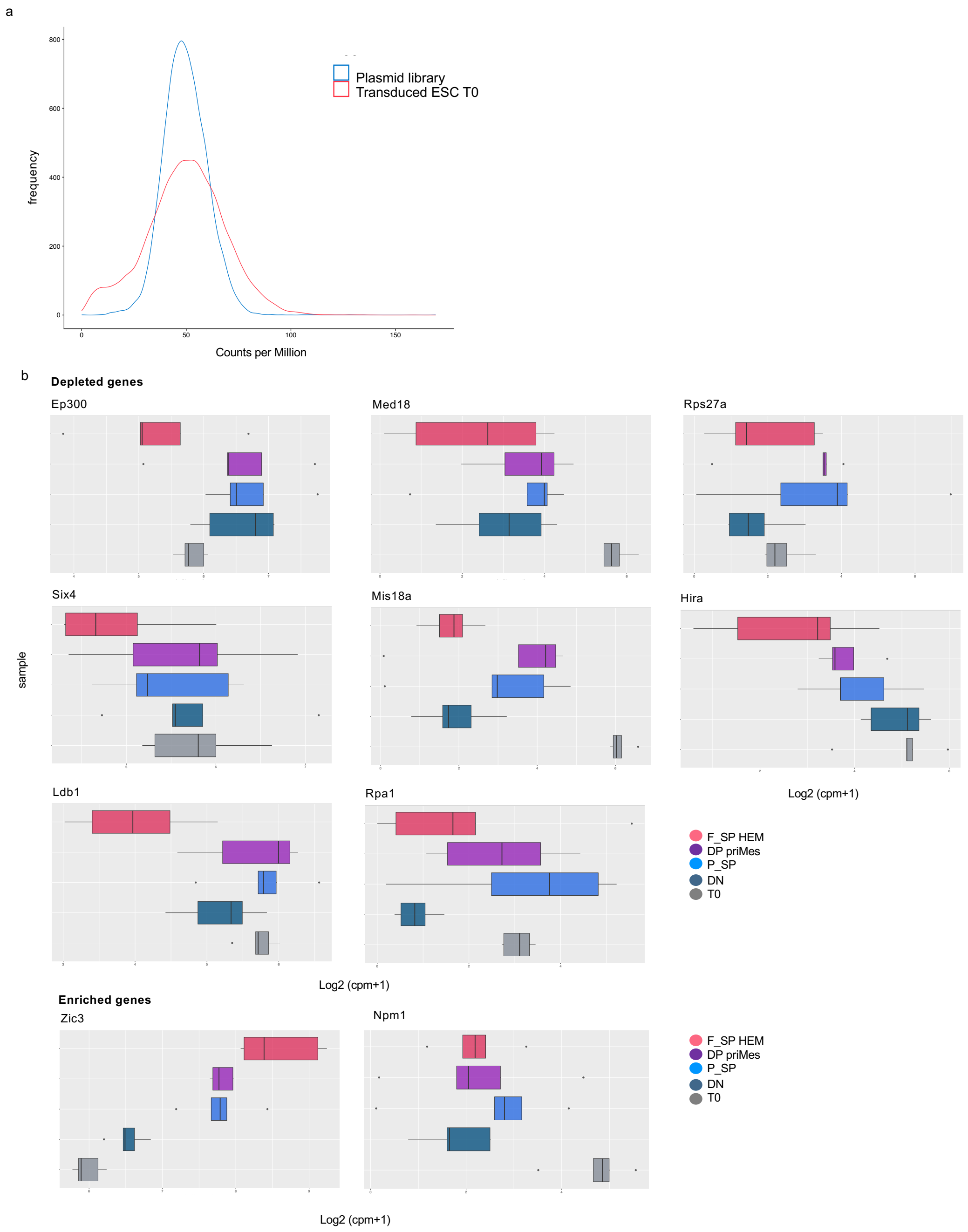

Supplementary Figure 5:

a Counts per million (CPM) values of sgRNA abundance of CRISPR-Cas9 library representation of the original library (blue) and at day 0 after transduction of ESCs at the start of EB cultures (red). **b** Representative counts per million (CPM) values of sgRNA abundance at T0 and mesodermal populations of indicated genes. Box plot represents upper and lower quartiles. Whiskers extend to the smallest and largest observed values within Q1 − 1.5 × IQR and Q3 + 1.5 × IQR; values beyond these limits are plotted as outliers. Central line is the median.n=3 biological replicates.

a

depleted genes	rank	p-value
Smad1	1	4.47E-15
Etv2	2	4.88E-07
Ep300	3	7.27E-07
Rps27a	4	2.39E-06
Mis18a	5	2.60E-08
Ldb1	6	9.72E-06
Rpa1	7	3.86E-06
Med18	8	2.09E-05
Six4	9	0.0001064
Hira	15	0.0002898
**enriched genes**		
Cop1	1	2.71E-09
Zbtb7b	2	7.65E-06
Zic3	3	2.00E-06
Npm1	4	1.54E-04

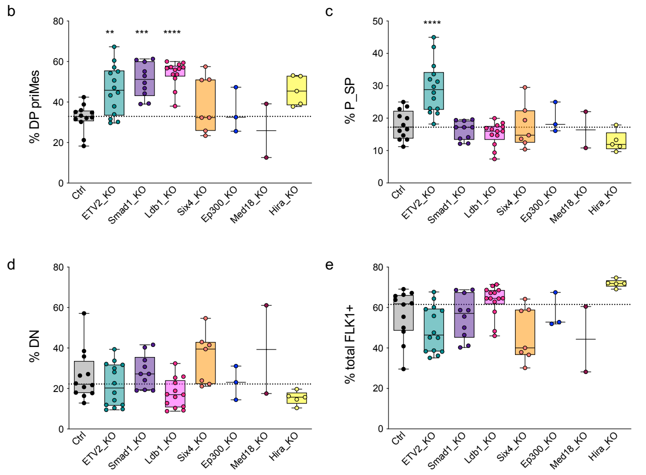

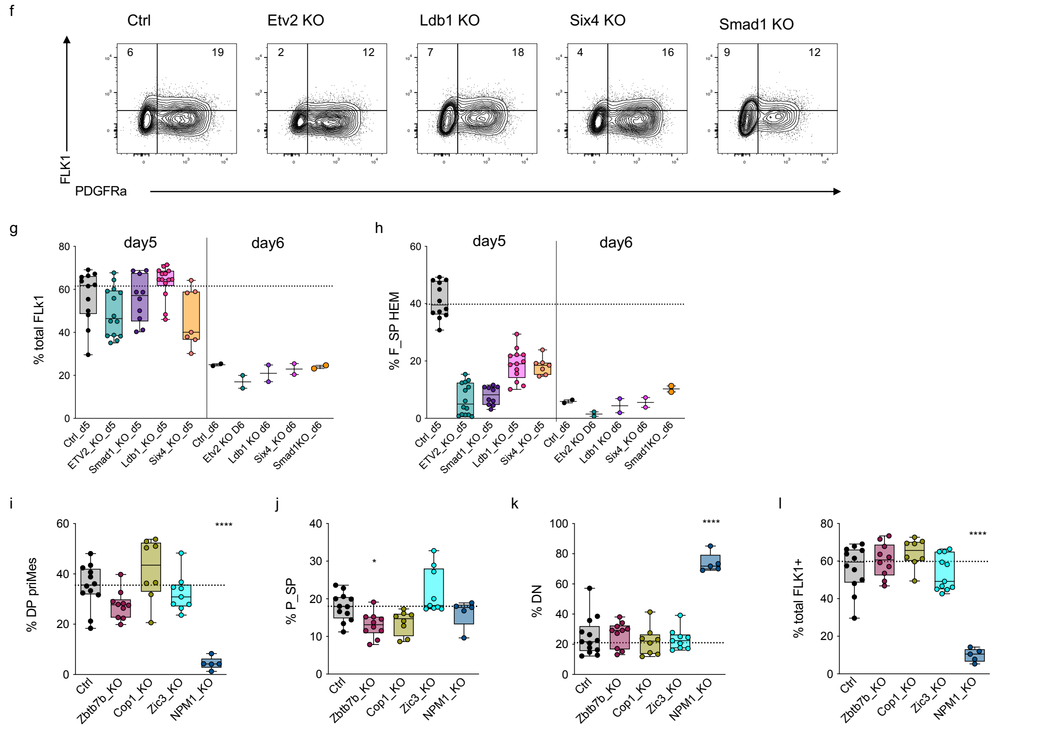

Supplementary Figure 6:
a Table of candidate genes that were used for validation experiments of depleted and enriched genes. Frequency of (**b**) DP priMes (Ctrl vs. *ETV2* KO p= 0.0049, Ctrl vs. *Smad1* KO p=0.0002, Ctrl vs. *Ldb1* KO p< 0.0001), (**c**) P_SP (Ctrl vs. *ETV2* KO p< 0.0001) (**d**) DN and (**e**) total FLK1in EB cultures day 5 of indicated KOs. **f** Representative flow cytometry analysis of FLK1 and PDGFRα surface expression in EB cultures from indicated KO cells at day 6. Flow cytometry analysis of (**g**) total FLK1 and (**h**) frequency of F_SP HEM of EB cultures from indicated KO cells at day 5 and day 6. Frequency of (**i**) DP priMes (Ctrl vs. *Npm1* KO p< 0.0001), (**j**), P_SP (Ctrl vs. *Zbtb7b* KO p= 0.0338) (**k**) DN (Ctrl vs. *Npm1* KO p< 0.0001) and (**l**) total FLK1 (Ctrl vs. *Npm1* KO p< 0.0001) in EB cultures day 5 of indicated KOs. Each data point (in b-e, g-l) represents an individually generated culture. For each cell line 3 independent clones were analysed, n = 4 biological replicates. P-values were calculated using a one-way ANOVA multiple comparisons analysis. Box plot whiskers represent the minimum and maximum value. Bounds mark the 25th and 75th percentiles. Central line is the median. Source data for (**b-e, g-l**) are provided as a Source Data file.

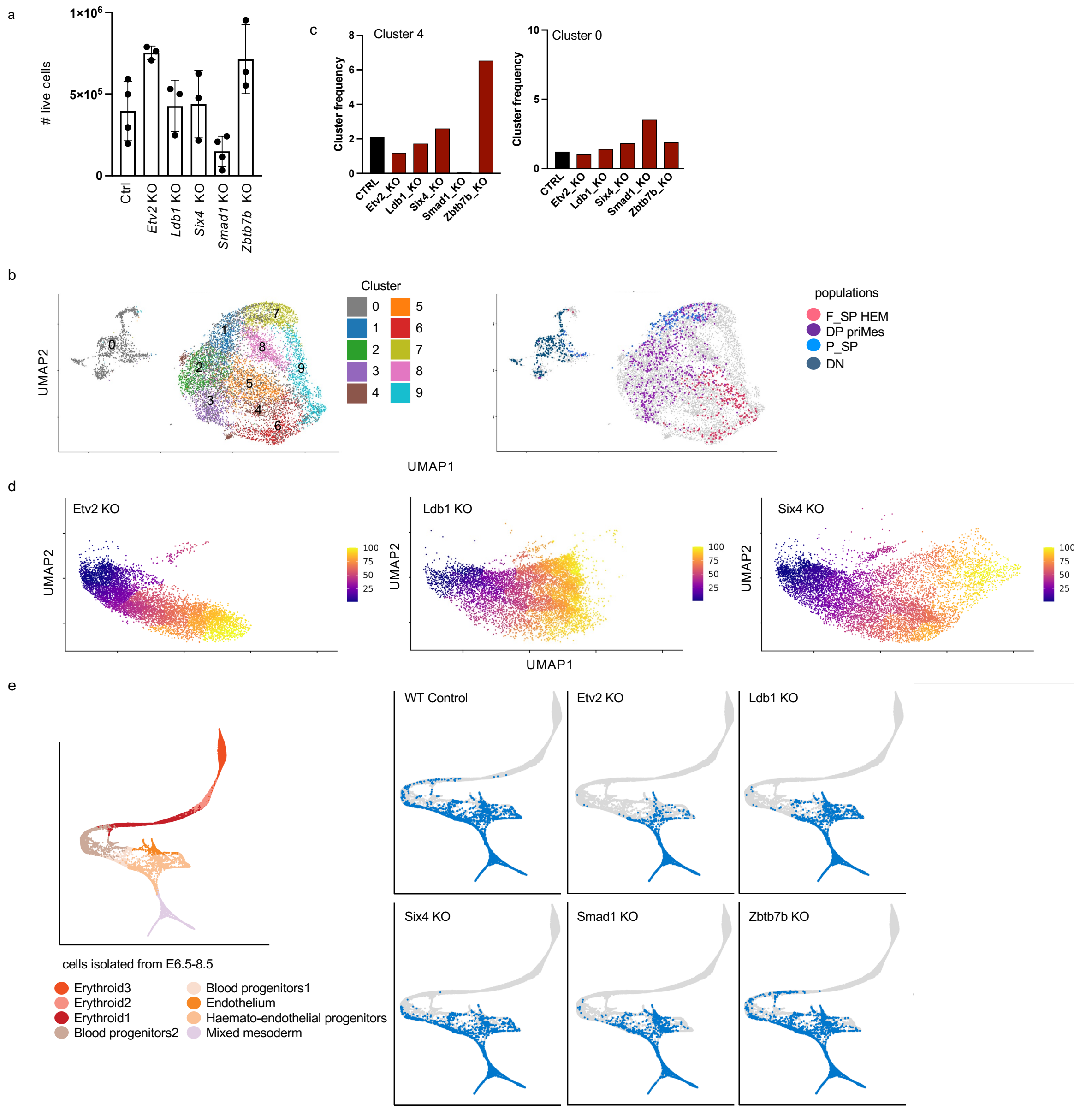

Supplementary Figure 7:
a Number of live cells in haemangioblast cultures of indicated KOs. Each data point represents an individually generated culture. For each cell line three independent KO clones were analysed. Error bars represent mean +/− SD n=3 biological replicates. **b** UMAP visualizing the integration of both scRNA-seq datasets from Fig 1. and Fig.5. (Left) cells coloured by clusters and (right) highlighting the cell surface expression of WT scRNA-seq samples from Fig.1. **c** Frequency of Cluster 4 and Cluster 0 in the indicated genotypes. **d** UMAP visualizing the pseudotime trajectory of indicated genotypes.**e** (left) Force- directed graph layout of cells isolated from E6.5–E8.5 embryos associated with the blood/endothelial lineage (adapted from[21]) (see Fig. 1f. Mapping of FACS sorted FLK1+ cells (in blue) isolated from day 5 EB cultures for indicated genotypes onto the blood-related differentiation trajectory (grey)[21]. Source data for (**a, c**) are provided as a Source Data file.

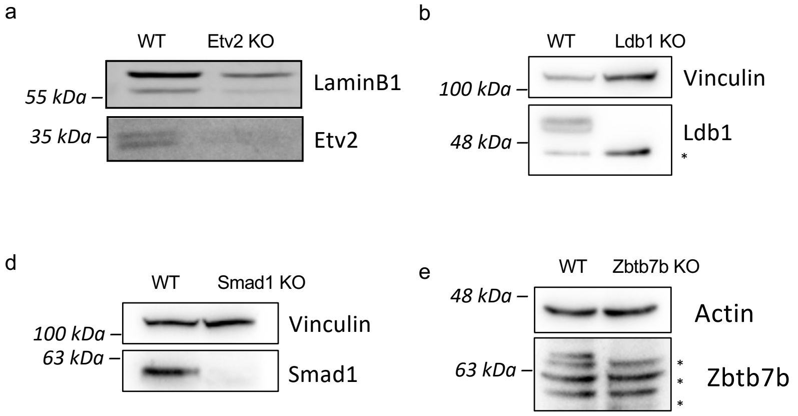

Supplementary Figure 8:
Western blot validation of indicated KO lines using nuclear extracts of EB cultures day 5 from WT and **a** *Etv2* KO, **b** *Ldb1* KO, **c** *Six4* KO, **d** *Smad1* KO and **e** *Zbtb7b* KO probed with antibodies against ETV2, LDB1, SIX4, SMAD1 and ZBTB7B respectively. Lamin B1, vinculin, tubulin and β-actin act as loading controls. Asterisks denote unspecific bands. Approximate sizes are indicated in kDa. Experiment was performed once.

Supplementary Table 1: Antibody panel for spectral flow analysis of haemangioblast cultures

antibody	comments/ marker specificity
CD117 (cKit)	haematopoietic cells, primitive haematopoietic cells: cKit neg; definitive haematopoietic cells: cKit pos
CD11b	haematopoietic cells, myeloid and immune cells
CD11c	haematopoietic cells, myeloid and immune cells
CD14	myeloid cells, low to variable expression in primitive macrophages/ tissue resident macrophages
CD16_32	myeloid and immune cells
CD202b (Tie2)	endothelial cells, HSPCs
CD309 (Flk1)	endothelial progenitors, haematopoietic-endothelial mesoderm (haemangioblast), mesodermal progenitors
CD31 (Pecam1)	mature/ differentiated endotheial cells
CD34	endothelial cells, HSPCs
CD41	early haematopoietic cell marker, most reluable marker for early haematopoietic cells in yolk sac
CD43	early haematopoietic cell marker
CD44	haematopoietic cells, subset of VSM cells
CD45	pan-leukocyte marker, expressed on primitive macrophages
CD47	ubiquitinous expression
CD64	activated macrophages and monocytes
CD71	erythroid progenitors
CD9	platelets, haematopoietic stem cells, various immune cells, epithelial cells
CD93	endothelial cells, early haematopoietic progenitors, myeloid cells
Cx3CR1	monocytes and macrophage marker
CD326 (Epcam)	EpiSC marker, epithelial cells
Ly6C	monocyte subsets
Sca1	HSPCs at later time points, not expressed in early HSPC of the yolk sac
CD144 (VE-Cadherin)	vascular enothelial cells, most reliable vascular endothelial marker

Supplementary Table 2: scRNA-seq Cluster Markers

Cl1	Cl2	Cl3	Cl4	Cl5	Cl6	Cl7	Cl8	Cl9	Cl0
Mesp1	Dkk1	Krt18	Lmo2	Exoc3l	Prkar2b	Sfrp1	Optc	Ctla2a	Trh
Tdgf1	Foxf1	Col1a1	Fgf3	Etv2	Fli1	Foxc2	Tll1	Plxnd1	Mt2
Frzb	Tbx2	Hand1		Nrp2	Fgf3	Nnat	Cyp26a1	Rasip1	Cdh1
Lhx1	Msx1	Krt8		Pdlim3	Gadd45g	Dll1	Car2	Rhoj	Igfbp3
Mixl1	Bmp4	Ahnak		Lmo2	Lmo2	Dll3	Bmp2	Ramp2	Mt1
Snai1	Ube2c	Foxf1		Mmp9	F2r	Foxc1	Ctsc	Gadd45g	Cldn6
Sp5	Lmo1	Mest		Lratd2	Hbb-bh1	Lrig3	Tdgf1	Exoc3l	Chchd10
Cachd1	Amer3	Msx2		Mixl1	Tal1	Crabp1	Cbln1	Trp53i11	Bst2
Eomes	Pmp22	Stard8		Gata3	Gata2	Lfng	Fgf10	Flt1	Cldn7
Upp1	Cenpf	Peg10		Pcdh1	Efnb2	Pcdh8	Gpc4	Flt4	Epcam
Lmo1	Hand1	Pard6b		Tal1	Sox7	Trp53i11	Etv2	Col4a1	Nanog
Fst	Cfc1	Tmem88		Nav1	Phf6	Lhfp	Igfbp4	Sox7	Slc2a3
Arl4d	Nusap1	Csrp2		Nxph4	Rnd2	Pkdcc	Eomes	Shank3	Sox2
Efna3	Vldlr	Spin2c		E2f4	Cbfa2t3	Lhx1	Frmd4b	Atf3	Utf1
Dlc1	Vstm2b	Unc5c		Cd40	Phlda1	Jag1	Otx2	Septin8	Pim2
Pid1	Olfm1	Dok4		Fam43a	Gclm	Epb41l3	Lhx1	Tll1	Kif1a
Pmaip1	Plxna4	Bmp4		T	Hhex	Col23a1	Trib1	Cdh5	L1td1
Lefty2	Bambi	Foxo4		Amot	Wnk4	Zic3	Zic2	Sox18	Esrp1
Fgf8	Tmem88	Bex1		Shank3	Egfl7	Cer1	Adcyap1r1	Cyp26a1	Spint2
Evx1	Plk1	Arl4c		Eng	Lgr5	Lama1	Sox9	Egfl7	Apela
Pdgfra	Ptpn13	Tbx2		Vax1	Tnfaip2	Aplnr	Cdh2	Hhex	St14
Dusp6	Zfp703	Car4		Crym	Inka1	Adam19	Lrig3	Pkdcc	Cldn4
Otx2	Six2	Slc2a1		Sgk3	Rbm38	Frzb	Map1b	Fli1	Camsap3
Emb	Arl6ip1	Akap12		Tbc1d14	Myb	Emb	Adam19	Tal1	Tdh
Cabp1	Trpc3	Nkd1		Magi3	Etl4	Epha4	Vax1	Col18a1	Nodal
Dll3	Cxcr4	Tbx20		Car2	Ets2	Plxna2	Dusp6	Elk3	Fgf5
Zic2	Msx2	Slc38a4		Tgfb2	Runx1	Tenm4	Dlc1	Col4a2	F2rl1
Gsc	Hmmr	Pmp22		Adra2b	Nfxl1	Irx3	Nrp2	Calcrl	B3gnt7
Dpysl5	Prex1	Morc4		Enc1	Rasip1	Ifitm1	Fzd7	Tmsb4x	Gbp2
Sgk1	Eomes	Tpm1		Zfp703	Tmem38b	Dact1	Robo1	Igfbp4	Gldc
Wnt5a	Bmper	Fgfr2		Lifr	Irs2	Cd24a	Septin8	Bmp2	Pdpn
Ypel3	Wnt5a	Phlda2		Ass1	Abcb10	Hoxb1	Mthfd2	Tgfbr3	Bex1
Sall4	Slc6a6	Zfp703		Cnn2	Myo10	Rimbp2	Myl7	Tspan18	Ezr
Etv5	Nog	Crb2		Ndufa4l2	Igf2	Otx2	Col13a1	Tek	Fgf8
Pcdh7	Vrtn	Mmp15		Cnksr3	Ramp2	Sulf1	Hmga2	Ppp1r13b	Epha2
Pitx2	Ccnf	Podxl		Fhod1	Slc1a3	Sox4	Mmp9	Phlda1	Plekhf2
Epha1	Aurka	Capn6		Nectin2	Zfp711	Wnt2b	Gsc	F2r	Fabp3
Dock11	Aspm	Tnfrsf19		Id4	Slc39a8	Septin8	Mixl1	Arap3	Igfbp2
Rbp1	Fzr1	Gjb2		Slc39a4	Slc29a1	Myl7	Crabp1	Hapln1	Epha1
Robo1	Pcdh7	Wnt6		Ctsc	Aff3	Laptm4b	Spry4	Tnfaip2	Trp53inp1
Smad1	Cdc20	Cdh11		Id3	Stat5b	Dapk1	Emb	St8sia1	Dnmt3b
Phlda2	Evx1	Peg3		Kctd12	Fth1	Asb4	Nxph4	Itm2a	Stmn2
Six2	Tbx3	Rbpms2		F11r	Smtnl2	Tubb2a	Dapk1	Foxc2	Mkrn1
Mesp2	Fam122b	Pdlim3		Slc43a1	Itm2a	Sms	Sall1	Enc1	Irf1
Pbx1	L1cam	Cdkn1c		Atp8a1	Adam23	Ldhb	Mpped2	Selenop	Zfp42
Dact1	Top2a	Cfc1		Tjp2	Rspo3	Fzd2	Pawr	Gngt2	Krt19
Mycn	St6galnac4	Bcam		Elk3	Slc2a3	Sall1	Asns	Etv2	Dppa5a
Trib2	Cabp1	Ugdh		Thy1	Pde4dip	Tubb2b	Flnb	Abi3	Fst
Hs3st3b1	Ndufa4l2	Pwwp3b		Rgs5	Nrip3	Irx1	Gata6	Lmo2	Sox17
Fzd7	Hand2	Anxa2		Dusp6	Gfi1b	Mesp2	Car4	Dysf	T

Supplementary Table 3: Targeting sgRNA sequences

Gene	sgRNA 1	sgRNA 2	targeting region	size of deletion (bp)
Smad1	CATGAGGTAGTCCCTTCCGG	CCCCTACCACTATAAGCGAG	Exon 2	152 bp
Etv2	TGGAAAGGTACGTCTTCGTG	AGTCTCGCGACCACGGAAGG	Exon 3 / Exon 4	259 bp
Ep300	GAATTGGGACTAACCAATGG	TAGCATGGTCAAAAGCCCAA	Exon 2	203 bp
Rps27a	GGGGAAAACCATCACGCTCG	CGAGTGGCGAAATGTAGGGT	Exon 2 / Intron 2-3	159 bp
Mis18a	AAGACTCGAGCCGCTACCTG	ACTGGGCGACTCGCTCACCT	Exon 1	145 bp
Ldb1	GAGCACGTAGTACAGCTCTG	CGACTGTGACCAGGGCAGCA	Exon 6	61 bp
Rpa1	ATGGTGGGACACCTGAGCGA	GGCCTTGGAGCGGTGCAAAG	Exon 1 / Intron 1-2	76 bp
Med18	GGAGAAGCACAGGATCCACC	ATGATGGAGTACCTCCTGCA	Exon 2	143 bp
Six4	AGGGGCACTAGGGCTGTAGG	GAAGCTGCTACTGAGACAGG	Exon 2	221 bp
Cop1	AGTTCATTCACTGAAAACAA	CAATGCTGGAGATTGAATCA	Exon 4 / Intron 4-5	151 bp
Zbtb7b	GCTGGACTTTGTAGGGCCAG	GCTTGCATGGAGATTCTACA	Exon 2	136 bp
Zic3	CAACCACGTCTGCTATTGGG	GATCTTGAGGTTCTCAGAGC	Exon 1	123 bp
Npm1	TCACAGGTCAGTTTAGGAGC	TATTTTTAGGTTTCCCTAGG	Exon 3 / Exon 4	301 bp

ANNEX

Uncropped WB scan of Supplementary Figure 8:

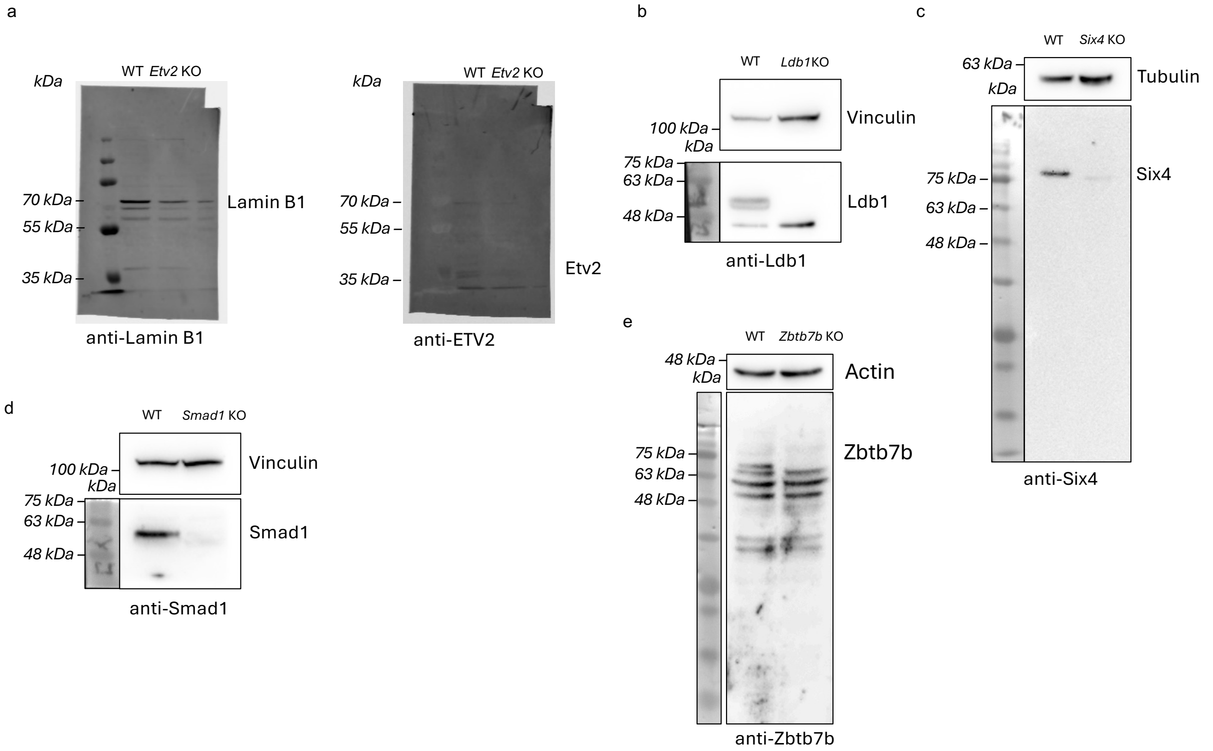

Supplement: Supplementary file 1 — Supplementary Information [file 41467_2025_66230_MOESM1_ESM.pdf]
