# Supplementary material for: Targeted CRISPR-Cas9 screening identifies core transcription factors controlling murine haemato-endothelial fate commitment
Source: Nat Commun. 2025 Dec 13;16:11412. doi: 10.1038/s41467-025-66230-9 (PMC12738756; doi:10.1038/s41467-025-66230-9)
Supplement: Supplementary file 2 — Description of Additional Supplementary Files [file 41467_2025_66230_MOESM2_ESM.pdf]

Description of Additional Supplementary Files

File Name: Supplementary Data 1

Description: Excel table Overlaps_all_transitions_with rank
