# Supplementary material for: Targeted CRISPR-Cas9 screening identifies core transcription factors controlling murine haemato-endothelial fate commitment
Source: Nat Commun. 2025 Dec 13;16:11412. doi: 10.1038/s41467-025-66230-9 (PMC12738756; doi:10.1038/s41467-025-66230-9)
Supplement: Supplementary file 4 — Reporting Summary [file 41467_2025_66230_MOESM4_ESM.pdf]

nature portfolio

Corresponding author(s): Nina Schmolka

Last updated by author(s): Oct 10, 2025

Reporting Summary

Nature Portfolio wishes to improve the reproducibility of the work that we publish. This form provides structure for consistency and transparency in reporting. For further information on Nature Portfolio policies, see our Editorial Policies and the Editorial Policy Checklist.

Statistics

For all statistical analyses, confirm that the following items are present in the figure legend, table legend, main text, or Methods section.

n/a	Confirmed	
☐	☒	The exact sample size (*n*) for each experimental group/condition, given as a discrete number and unit of measurement
☐	☒	A statement on whether measurements were taken from distinct samples or whether the same sample was measured repeatedly
☐	☒	The statistical test(s) used AND whether they are one- or two-sided<br>*Only common tests should be described solely by name; describe more complex techniques in the Methods section.*
☒	☐	A description of all covariates tested
☐	☒	A description of any assumptions or corrections, such as tests of normality and adjustment for multiple comparisons
☐	☒	A full description of the statistical parameters including central tendency (e.g. means) or other basic estimates (e.g. regression coefficient) AND variation (e.g. standard deviation) or associated estimates of uncertainty (e.g. confidence intervals)
☐	☒	For null hypothesis testing, the test statistic (e.g. *F*, *t*, *r*) with confidence intervals, effect sizes, degrees of freedom and *P* value noted<br>*Give P values as exact values whenever suitable.*
☒	☐	For Bayesian analysis, information on the choice of priors and Markov chain Monte Carlo settings
☒	☐	For hierarchical and complex designs, identification of the appropriate level for tests and full reporting of outcomes
☒	☐	Estimates of effect sizes (e.g. Cohen's *d*, Pearson's *r*), indicating how they were calculated

Our web collection on statistics for biologists contains articles on many of the points above.

Software and code

Policy information about availability of computer code

Data collection	For data collection we used standard operating software installed on Illumina Nova Sequencer, BD Biosciences Symphony cytometer and BD Biosciences Aria III cell sorter, Cytek Aurora.
Data analysis	For data analysis the following publicly available tools have been use:<br>STAR (2.7.7)<br>STARSolo (2.7.3a)<br>scater (1.26)<br>Seurat (4.3)<br>scvelo (0.2.5)<br>CellRanger Multi (7.2.0)<br>Monocle3 (1.0.0)<br>bcl2fastq2 (2.20.0.422)<br>cutadapt (3.1)<br>bowtie2 (2.3.5)<br>MAGeCK (0.5.9)<br>FlowJo (10.7)<br>BD FACS Diva (9.1.2)<br>R (4.1.2)<br>Prism (5.0a)<br>FlowSom (2.17.0)

For manuscripts utilizing custom algorithms or software that are central to the research but not yet described in published literature, software must be made available to editors and reviewers. We strongly encourage code deposition in a community repository (e.g. GitHub). See the Nature Portfolio guidelines for submitting code & software for further information.

Data

Policy information about availability of data

All manuscripts must include a data availability statement. This statement should provide the following information, where applicable:

- Accession codes, unique identifiers, or web links for publicly available datasets
- A description of any restrictions on data availability
- For clinical datasets or third party data, please ensure that the statement adheres to our policy

Newly generated scRNAseq data sets have been deposited to NCBI GEO under the following accession number: GSE287036 and GSE302880. The newly-generated bulk RNAseq dataset under: GSE302791.

Research involving human participants, their data, or biological material

Policy information about studies with human participants or human data. See also policy information about sex, gender (identity/presentation), and sexual orientation and race, ethnicity and racism.

Reporting on sex and gender	NA
Reporting on race, ethnicity, or other socially relevant groupings	*Please specify the socially constructed or socially relevant categorization variable(s) used in your manuscript and explain why they were used. Please note that such variables should not be used as proxies for other socially constructed/relevant variables (for example, race or ethnicity should not be used as a proxy for socioeconomic status).*<br>*Provide clear definitions of the relevant terms used, how they were provided (by the participants/respondents, the researchers, or third parties), and the method(s) used to classify people into the different categories (e.g. self-report, census or administrative data, social media data, etc.)*<br>*Please provide details about how you controlled for confounding variables in your analyses.*
Population characteristics	*Describe the covariate-relevant population characteristics of the human research participants (e.g. age, genotypic information, past and current diagnosis and treatment categories). If you filled out the behavioural & social sciences study design questions and have nothing to add here, write "See above."*
Recruitment	*Describe how participants were recruited. Outline any potential self-selection bias or other biases that may be present and how these are likely to impact results.*
Ethics oversight	*Identify the organization(s) that approved the study protocol.*

Note that full information on the approval of the study protocol must also be provided in the manuscript.

Field-specific reporting

Please select the one below that is the best fit for your research. If you are not sure, read the appropriate sections before making your selection.

☒ Life sciences ☐ Behavioural & social sciences ☐ Ecological, evolutionary & environmental sciences

For a reference copy of the document with all sections, see nature.com/documents/nr-reporting-summary-flat.pdf

Life sciences study design

All studies must disclose on these points even when the disclosure is negative.

Sample size	No statistical method was used to pre-determin sample size.
Data exclusions	na
Replication	All experiments were confirmed by replication at least once. For each mutant cell line three independent clones were generated. The exact handling of replicates is depicted in figure panels or the method section.
Randomization	Not relevant for the experiments performed here. Samples were allocated either wild type control or mutant and processed in parallel.
Blinding	Blinding not relevant for this study. All samples processed through identical analysis pipelines in parallel.

Reporting for specific materials, systems and methods

We require information from authors about some types of materials, experimental systems and methods used in many studies. Here, indicate whether each material, system or method listed is relevant to your study. If you are not sure if a list item applies to your research, read the appropriate section before selecting a response.

Materials & experimental systems

n/a	Involved in the study	
☐	☒	Antibodies
☐	☒	Eukaryotic cell lines
☐	☐	Palaeontology and archaeology
☐	☐	Animals and other organisms
☐	☐	Clinical data
☐	☐	Dual use research of concern
☐	☐	Plants

Methods

n/a	Involved in the study	
☐	☐	ChIP-seq
☐	☒	Flow cytometry
☐	☐	MRI-based neuroimaging

Antibodies

Antibodies used

The following antibodies were used for conventional flow cytometry analysis:
anti-CD309 (FLK1) (APC, BD Bioscience, clone Avas12a1) 1:200
anti-CD140a (PDGFR) (PE, BD Bioscience, clone APA5) 1:200
anti-CD41 (BV650, BD Biosciences, Clone MWReg30) 1:200
anti-VE-cadherin (eFluor 660, BD Biosciences clone eBioBV13) 1:200
anti-ckit (PE-Cyanine7, BD Bioscience, clone ACK2) 1:200
anti-CD45 (PerCPCy5.5, BD Biosciences, clone 30F-11) 1:200
anti-CD71 (RB780, BD Biosciences, clone C2) 1:200

The following antibodies were used for spectral flow cytometry analysis:

From BD biosciences:
anti-CD117(cKit) (BUV395, Clone 2B8) 1:200,
anti-CD43 (BUV496, Clone 1B11) 1:400
anti-CD45 (BUV563, Clone 30-F11) 1:200
anti-CD9 (BUV661, Clone KMC8) 1:500
anti-CD44 (BUV737, Clone IM7)1:500
anti-CD31 (BUV805, Clone 390) 1:400
anti-Epcam (CD326) (BV480, Clone G8.8)1:200
anti-CD93 (BV605, Clone AA4.1) 1:600
anti-CD41 (BV650, Clone MWReg30) 1:600
anti-CD14 (FITC, Clone rmC5-3) 1:200
anti-CD71 (RB780, Clone C2) 1:1400
anti-CD47 (PE-CF594, Clone miap301) 1:200.
From BioLegend:
anti-CD64 (BV421, Clone X54-5/7.1) 1:200
anti-CD16/32 (BV711, Clone 93) 1:1200
anti-CX3CR1 (BV785, Clone SA011F11)1:400
anti-CD202b (Tie2) (PE, Clone TEK4) 1:1000
anti-CD34 (PE-Cy5, Clone MEC14.7) 1:400
anti-CD309 (FLK1) (PE-Cy7, Clone Avas12) 1:400
anti-Ly6C (Alexa Fluor 700, Clone HK1.4) 1:1000
anti-Sca-1 (APC-Fire 750, Clone D7) 1:1000
anti-CD11b (APC-Fire 810, Clone M1/70) 1:400

From ThermoFisher:
anti-CD11c (PE-Cy5.5, Clone N418) 1:400
anti-VE-cadherin (CD144) (eFluor660, Clone BV13) 1:900

The following antibodies were used for WB:
anti-Lamin B1, sc-374015, Santa Cruz Biotechnology) 1:1000
anti-Six4 (Clone: D-5), sc-390779, Santa Cruz Biotechnology) 1:250
anti-Zbtb7b (TH-POK) (Clone: A-4), sc-376250, Santa Cruz Biotechnology) 1:50
anti-Ldb1 (Clim1/2) (Clone:A-3), sc-376030, Santa Cruz Biotechnology) 1:500
anti-Smad1 (Clone:A-4), sc-7965, Santa Cruz Biotechnology)1:500
anti-ETV2, (Clone:EPR5229(3)), abcam)1:500

Validation

all antibodies are commercial antibodies and validated by commercial provider and through numerous publications.

Eukaryotic cell lines

Policy information about cell lines and Sex and Gender in Research

Cell line source(s)	murine embryonic stem cells (HA36CB1, 129xC57Bl/6) Baubec et al. DOI:101016/j.cell.2013.03.011
Authentication	Genotype confirmed by PCR/Sanger sequencing and western blot.
Mycoplasma contamination	Tested negative

Commonly misidentified lines (See ICLAC register)	No commonly misidentified cell lines were used.

Palaeontology and Archaeology

Specimen provenance	NA
Specimen deposition	*Indicate where the specimens have been deposited to permit free access by other researchers.*
Dating methods	*If new dates are provided, describe how they were obtained (e.g. collection, storage, sample pretreatment and measurement), where they were obtained (i.e. lab name), the calibration program and the protocol for quality assurance OR state that no new dates are provided.*

☐ Tick this box to confirm that the raw and calibrated dates are available in the paper or in Supplementary Information.

Ethics oversight	*Identify the organization(s) that approved or provided guidance on the study protocol, OR state that no ethical approval or guidance was required and explain why not.*

Note that full information on the approval of the study protocol must also be provided in the manuscript.

Animals and other research organisms

Policy information about studies involving animals; ARRIVE guidelines recommended for reporting animal research, and Sex and Gender in Research

Laboratory animals	NA
Wild animals	*Provide details on animals observed in or captured in the field; report species and age where possible. Describe how animals were caught and transported and what happened to captive animals after the study (if killed, explain why and describe method; if released, say where and when) OR state that the study did not involve wild animals.*
Reporting on sex	*Indicate if findings apply to only one sex; describe whether sex was considered in study design, methods used for assigning sex. Provide data disaggregated for sex where this information has been collected in the source data as appropriate; provide overall numbers in this Reporting Summary. Please state if this information has not been collected. Report sex-based analyses where performed, justify reasons for lack of sex-based analysis.*
Field-collected samples	*For laboratory work with field-collected samples, describe all relevant parameters such as housing, maintenance, temperature, photoperiod and end-of-experiment protocol OR state that the study did not involve samples collected from the field.*
Ethics oversight	*Identify the organization(s) that approved or provided guidance on the study protocol, OR state that no ethical approval or guidance was required and explain why not.*

Note that full information on the approval of the study protocol must also be provided in the manuscript.

Clinical data

Policy information about clinical studies
All manuscripts should comply with the ICMJE guidelines for publication of clinical research and a completed CONSORT checklist must be included with all submissions.

Clinical trial registration	NA
Study protocol	*Note where the full trial protocol can be accessed OR if not available, explain why.*
Data collection	*Describe the settings and locales of data collection, noting the time periods of recruitment and data collection.*
Outcomes	*Describe how you pre-defined primary and secondary outcome measures and how you assessed these measures.*

Dual use research of concern

Policy information about dual use research of concern

Hazards

Could the accidental, deliberate or reckless misuse of agents or technologies generated in the work, or the application of information presented in the manuscript, pose a threat to:

No	Yes	
☒	☐	Public health
☒	☐	National security
☒	☐	Crops and/or livestock
☒	☐	Ecosystems
☒	☐	Any other significant area

Experiments of concern

Does the work involve any of these experiments of concern:

No	Yes	
☒	☐	Demonstrate how to render a vaccine ineffective
☒	☐	Confer resistance to therapeutically useful antibiotics or antiviral agents
☒	☐	Enhance the virulence of a pathogen or render a nonpathogen virulent
☒	☐	Increase transmissibility of a pathogen
☒	☐	Alter the host range of a pathogen
☒	☐	Enable evasion of diagnostic/detection modalities
☒	☐	Enable the weaponization of a biological agent or toxin
☒	☐	Any other potentially harmful combination of experiments and agents

Plants

Seed stocks: NA

Novel plant genotypes: *Describe the methods by which all novel plant genotypes were produced. This includes those generated by transgenic approaches, gene editing, chemical/radiation-based mutagenesis and hybridization. For transgenic lines, describe the transformation method, the number of independent lines analyzed and the generation upon which experiments were performed. For gene-edited lines, describe the editor used, the endogenous sequence targeted for editing, the targeting guide RNA sequence (if applicable) and how the editor was applied.*

Authentication: *Describe any authentication procedures for each seed stock used or novel genotype generated. Describe any experiments used to assess the effect of a mutation and, where applicable, how potential secondary effects (e.g. second site T-DNA insertions, mosiacism, off-target gene editing) were examined.*

ChIP-seq

Data deposition

☐ Confirm that both raw and final processed data have been deposited in a public database such as GEO.

☐ Confirm that you have deposited or provided access to graph files (e.g. BED files) for the called peaks.

Data access links
May remain private before publication.: NA

Files in database submission: *Provide a list of all files available in the database submission.*

Genome browser session
(e.g. UCSC): *Provide a link to an anonymized genome browser session for "Initial submission" and "Revised version" documents only, to enable peer review. Write "no longer applicable" for "Final submission" documents.*

Methodology

Replicates: *Describe the experimental replicates, specifying number, type and replicate agreement.*

Sequencing depth: *Describe the sequencing depth for each experiment, providing the total number of reads, uniquely mapped reads, length of reads and whether they were paired- or single-end.*

Antibodies: *Describe the antibodies used for the ChIP-seq experiments; as applicable, provide supplier name, catalog number, clone name, and lot number.*

Peak calling parameters: *Specify the command line program and parameters used for read mapping and peak calling, including the ChIP, control and index files used.*

Data quality: *Describe the methods used to ensure data quality in full detail, including how many peaks are at FDR 5% and above 5-fold enrichment.*

Software | *Describe the software used to collect and analyze the ChIP-seq data. For custom code that has been deposited into a community repository, provide accession details.*

Flow Cytometry

Plots

Confirm that:

- [x] The axis labels state the marker and fluorochrome used (e.g. CD4-FITC).
- [x] The axis scales are clearly visible. Include numbers along axes only for bottom left plot of group (a 'group' is an analysis of identical markers).
- [x] All plots are contour plots with outliers or pseudocolor plots.
- [x] A numerical value for number of cells or percentage (with statistics) is provided.

Methodology

Sample preparation: Cells were harvested by trypsination and single-cell suspensions were obtained. For cell-surface staining, cells were incubated for 20-30min at 4 degrees with saturationg concentrations of antibodies. LIVE/DEAD Fixable Near-IR Dead Cell stain (L34975, Invitrogen) or Zombie NIR fixable viability dye (Biolegend) were used to discriminate cell viability.

Instrument: Symphony (BD biociences), Canto (BD Biosciences), FACSAria III cell sorter (BD Biosciences), Aurora (Cytek).

Software: Data was acquired with the BD FACSDiva Software (BD Biosciences) and analysed with FlowJo 10.7

Cell population abundance: Purity was assessed for all the samples after sorting.

Gating strategy: Gating was performed based on WT cells gated for single cells, using fowrad scatter areas (FSC-A) vs. side scatter area (SSC-A) followed by FSC-A vs. forward scatter height (FSC-H). Gating was done with the help of single-staining controls.

- [x] Tick this box to confirm that a figure exemplifying the gating strategy is provided in the Supplementary Information.

Magnetic resonance imaging

Experimental design

Design type: NA

Design specifications: *Specify the number of blocks, trials or experimental units per session and/or subject, and specify the length of each trial or block (if trials are blocked) and interval between trials.*

Behavioral performance measures: *State number and/or type of variables recorded (e.g. correct button press, response time) and what statistics were used to establish that the subjects were performing the task as expected (e.g. mean, range, and/or standard deviation across subjects).*

Acquisition

Imaging type(s): *Specify: functional, structural, diffusion, perfusion.*

Field strength: *Specify in Tesla*

Sequence & imaging parameters: *Specify the pulse sequence type (gradient echo, spin echo, etc.), imaging type (EPI, spiral, etc.), field of view, matrix size, slice thickness, orientation and TE/TR/flip angle.*

Area of acquisition: *State whether a whole brain scan was used OR define the area of acquisition, describing how the region was determined.*

Diffusion MRI: [ ] Used [ ] Not used

Preprocessing

Preprocessing software: *Provide detail on software version and revision number and on specific parameters (model/functions, brain extraction, segmentation, smoothing kernel size, etc.).*

Normalization: *If data were normalized/standardized, describe the approach(es): specify linear or non-linear and define image types used for transformation OR indicate that data were not normalized and explain rationale for lack of normalization.*

Normalization template: *Describe the template used for normalization/transformation, specifying subject space or group standardized space (e.g. original Talairach, MNI305, ICBM152) OR indicate that the data were not normalized.*

Noise and artifact removal: *Describe your procedure(s) for artifact and structured noise removal, specifying motion parameters, tissue signals and physiological signals (heart rate, respiration).*

Volume censoring | *Define your software and/or method and criteria for volume censoring, and state the extent of such censoring.*

Statistical modeling & inference

Model type and settings | *Specify type (mass univariate, multivariate, RSA, predictive, etc.) and describe essential details of the model at the first and second levels (e.g. fixed, random or mixed effects; drift or auto-correlation).*

Effect(s) tested | *Define precise effect in terms of the task or stimulus conditions instead of psychological concepts and indicate whether ANOVA or factorial designs were used.*

Specify type of analysis: ☐ Whole brain ☐ ROI-based ☐ Both

Statistic type for inference | *Specify voxel-wise or cluster-wise and report all relevant parameters for cluster-wise methods.*

(See Eklund et al. 2016)

Correction | *Describe the type of correction and how it is obtained for multiple comparisons (e.g. FWE, FDR, permutation or Monte Carlo).*

Models & analysis

n/a	Involved in the study
☐	☐ Functional and/or effective connectivity
☐	☐ Graph analysis
☐	☐ Multivariate modeling or predictive analysis

Functional and/or effective connectivity | *Report the measures of dependence used and the model details (e.g. Pearson correlation, partial correlation, mutual information).*

Graph analysis | *Report the dependent variable and connectivity measure, specifying weighted graph or binarized graph, subject- or group-level, and the global and/or node summaries used (e.g. clustering coefficient, efficiency, etc.).*

Multivariate modeling and predictive analysis | *Specify independent variables, features extraction and dimension reduction, model, training and evaluation metrics.*
